# Measurement of Total Free Iron in Soils by H_2_S Chemisorption and Comparison with the Citrate Bicarbonate Dithionite Method

**DOI:** 10.1155/2016/7213542

**Published:** 2016-12-25

**Authors:** Shui-Sheng Fan, Feng-Hsiang Chang, Hsin-Ta Hsueh, Tzu-Hsing Ko

**Affiliations:** ^1^Anxi College of Tea Science, Fujian Agriculture and Forestry University, Fuzhou 350002, China; ^2^Department of Leisure, Recreation and Tourism Management, Tzu Hui Institute of Technology, Pingtung 926, Taiwan; ^3^Sustainable Environment Research Laboratories, National Cheng Kung University, Tainan 701, Taiwan; ^4^Fujian Provincial Technology Development Base of Tea Industry, Anxi 362406, China

## Abstract

Free iron is one of the major analytical items for soil basic properties. It is also an important indicator for understanding the genesis of soil, soil classification, and soil distribution behavior. In this study, an alternative analytical method (chemisorption) based on thermodynamic knowledge was proposed for measurement of total free iron oxides in soils. Several representative soil samples belonging to alfisols, ultisols, inceptisols, and entisols were collected from Taiwan and tested by the chemisorption, and the estimated total free iron oxides were compared with those measured from the traditional citrate bicarbonate dithionite (CBD) method. Experimental results showed that the optimal operating temperature was found to be at 773 K and the carbon monoxide (CO) is the best gaseous reagent to promote the formation of FeS. The estimated total free iron oxides for soil samples determined from the chemisorption in the presence of CO were very close to those from the CBD technique. The result of regression indicates that the estimated total free iron is strongly correlated with the CBD-Fe content (*R*^2^ = 0.999) in the presence of CO.

## 1. Introduction

Iron is the third greatest metal element in soils. The iron content in soils may vary between one and several hundred grams per kilogram depending on the type of parent materials. There are sixteen iron oxides, oxide hydroxides, and hydroxides known in the environment to date. Among these iron oxides, Fe_2_O_3_ (*α* and *γ* form), Fe_3_O_4_, and FeOOH (*α* and *γ* form) are of interest and attracted the greatest amount of attention due to their well distribution in soils and industries. Hematite, *α*-Fe_2_O_3_, is the oldest known iron oxide and is widespread in soils and rocks. Hematite consists of layers of octahedral FeO_6_, which are connected by edge and are face-sharing and stacked perpendicular to* c* direction. Magnetite, Fe_3_O_4_, is one of the spinel structural groups. This type of iron oxide differs from most other iron oxides in that it contains both divalent iron and trivalent iron. Its formula is written as Y[XY]O_4_, where X = Fe(II), Y = Fe(III), and the brackets denote octahedral sites. The structure consists of octahedral and mixed tetrahedral/octahedral layers stacked along (1,1,1) direction. Goethite, *α*-FeOOH, consists of an HCP array of O^2−^ and OH^−^ stacked along the (1,0,0) direction with Fe(III) ions occupying half the octahedral interstices within a layer. It is the most common iron oxide in soils due to its high thermodynamic stability. Lepidocrocite, *γ*-FeOOH, is a layered compound. The structure consists of arrays of CCP anions (O^2−^/OH^−^) stacked along the (0,5,1) direction with Fe(III) ions occupying the octahedral interstices. Like goethite, lepidocrocite consists of double chains of Fe(O,OH)_6_ octahedral running parallel to the* c*-axis.

In soil science, iron species can be generally classified into several groups, including organically bounding iron, amorphous iron, free iron, and total iron. Free iron is defined as the iron coated/adsorbed on the surface of soil but not counting in the lattice structure of the soil. For the basic soil analysis, free iron is the major analytical item because it is an important indicator for understanding the genesis of soil, soil classification, and soil distribution behavior [1, 2]. For example, the ratio of the amorphous iron and free iron is an important indicator to distinguish the weathering extent of soil horizon. Deb was among the first to use sodium dithionite to measure the free iron oxide content of soils [3]. More recently, a series of modified procedures were presented and reported in the past decade [4–8]. Among the reported extraction procedures, the citrate bicarbonate dithionite (CBD) method has been recognized as the most commonly used procedure for determination of total free iron oxides in soils or sediments [9]. Additionally, hematite, goethite, lepidocrocite, ferrihydrite, and noncrystalline iron as well as organic-complexed iron can be exacted by the CBD procedure. Unfortunately, the high sodium salt content in the CBD extracts not only causes severe carry-over effect but also tends to clog the burner head during atomic absorption spectrometry (AAS) analysis. Aspiration of deionized water or dilute acids for a longer period between samples is thus needed for accurate analysis. To overcome these shortcomings, an alternative analyzed method was developed and assessed for the determination of total free iron contents of soils in this study. On the other hand, a series of the reactions between iron oxides and H_2_S were experimentally performed at high temperature and results showed that the high reactivity between iron oxides and H_2_S was observed in our previous literatures [10–12]. Accordingly, the main objective of this research is to determine the total free iron contents by the chemisorption approach based on the thermodynamic knowledge. In addition, a series of experimental factors were also considered and tested in order to find out the optimal results.

## 2. Experimental

### 2.1. Soils Sampling and Treatment

Six soil orders developed in different parent materials with a wide range of total iron oxide content were selected to represent the test sample in this study. After sampling, unwanted materials such as leaves and pebbles were removed from soils and then dried at ambient temperature for a week. Prior to use, soil samples were ground with an agate mortar and sieves to pass through a 50-mesh sieve to obtain the acquired particle size (approximately 0.296 mm).

### 2.2. Soil Samples Analyses

Soil properties consisting of free iron oxides, soil pH, organic matters, and soil texture as well as cation exchange capacity were taken into account. Particle size distribution was obtained by the pipette method after removal of carbonate, organic matters, and MnO_2_. Carbonate was removed by 1 M NaOAc with pH = 5 at 60°C and organic matters and MnO_2_ were digested by 30% H_2_O_2_ [13]. Soil pH value was measured on a mixture of 1 : 1 soil/deionized water by glass electrode [14]. Organic matter content was determined by the Walkley-Black wet oxidation method [15]. Cation exchange capacity (CEC) was determined by the ammonium acetate method [16].

### 2.3. Citrate Bicarbonate Dithionite Procedure (CBD Procedure)

The citrate bicarbonate dithionite (CBD) procedure is a widely acceptable extractive method for determination of total free iron [17]. The extraction procedure was described as follows:

- Weight 0.5 g of dried and sieved soil into 100 mL polypropylene centrifuge tube
- Adding 40 mL of 0.3 M sodium citrate and 5 mL of 1 M sodium carbonate
- Heating the mixture in a water bath at 80°C for 30 minutes
- Adding 0.5 g of sodium dithionite to the hot suspension and stirring frequently for 10 minutes
- Adding 10 mL sodium saturated sodium chloride and 10 mL acetone to enhance flocculation
- After cooling to ambient temperature, centrifugation of the mixture at 3500 rpm for 10 minutes, and the suspension is siphoned off into a 200 mL volumetric flask

The solid residue is washed with distilled water and steps (ii)–(vi) are systematically repeated to confirm that free iron is completely extracted. Then the suspension is added to the previous volumetric flask and filtered with a 0.2 *μ*m Teflon filter and the total suspension volume is made up to 150 mL. The filtered suspension was analyzed with an atomic absorption spectrometry for the determination of iron. Duplicate measurements were conducted for each sample.

### 2.4. Elemental Analysis

Elementar Vario EL III Heraeus CHNOS Rapid F002, equipped with a flash combustion furnace and thermal conductivity detector, was used for the sulfur determination.

### 2.5. Reagents

All the chemical reagents used in this study were obtained from Fluka and Aldrich and were of ACS grade.

### 2.6. Experimental Configuration and Design

**Figure 1:**
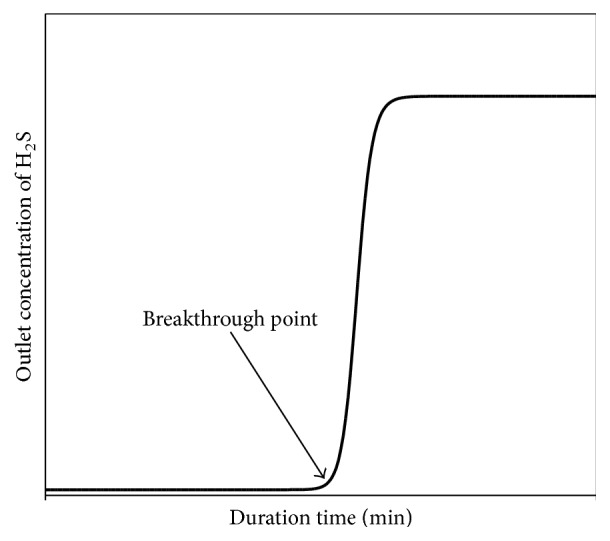
The typical H_2_S breakthrough curve and breakthrough point.

The chemisorption experiments were carried out in the fixed-bed reactor. All gases used in this study were prepared from regulation cylinders. Two sets of gases were prepared for the experiments. The mixture gases contained 50% CO and 1% H_2_S with balanced N_2_, and the other one contained 50% H_2_ and 1% H_2_S with balanced N_2_. Gas flow rates were monitored through mass flow controllers. All mass flow controllers were monitored accurately by an IR soap bubble meter and the concentration of all species calculated at the condition of standard temperature and pressure. The reactor consisted of a quartz tube, 1.6 cm i.d., 2.0 cm o.d., and 150 cm long, located inside an electric furnace. Quartz fibers were set in the reactor in order to support samples. Two K-type thermocouples were inserted exactly into the reactor near the positions on the top and bottom of the samples packing to measure and control the inlet and outlet temperatures. Prior to entering the reactor, the gases were conducted in a mixing pipe to confirm that the mixture gas was turbulent flow. Approximately 3 g of soil sample was weighted and placed into the quartz-made reactor. Before experiment proceeding, a pure nitrogen gas (purity 99.99%) was fed into the reactor for 30 minutes at 573 K in order to remove adsorbed water and impure materials coated on the surface of soil samples. In addition, blank experiments were also executed under the same conditions and verified that no reaction was taking place anywhere between H_2_S and the lines/reactor. The inlet and outlet concentration of H_2_S was analyzed by an on-line gas chromatograph equipped with a flame photometry detector. A six-port sampling with 0.5 mL sampling loop was used to sample the inlet and outlet concentration of H_2_S. The experiment was terminated when the outlet H_2_S concentration from the reactor approached the inlet concentration of H_2_S.  Figure 1 shows the typical breakthrough curve that derives from experiment in this study. When the mixture gases which contained 50% CO (or H_2_) and 1% H_2_S with balanced N_2_ were introduced into a reactor, the outlet concentration of H_2_S was systematically recorded. It is expected that the outlet concentration of H_2_S approached zero due to the complete reaction with free iron. The representative schemes are shown in the reactions of (1)–(4) (shown in Results and Discussion section). When the reaction between H_2_S and free iron was completed, H_2_S can be obviously detected. A breakthrough curve can be obtained by plotting H_2_S effluent concentration versus reaction time and a time at the breakthrough point is defined as breakthrough time. In this study, the breakthrough time was defined as the time from the beginning of the reaction to the point outlet H_2_S concentration reached 100 ppm. On the basis of breakthrough time and mass flow rate of H_2_S, the sulfur capacity can be accurately determined and thus the content of total free iron is estimated.

## 3. Results and Discussion

Two distinct adsorption mechanisms which are well discussed are physisorption (physical adsorption) and chemisorption (chemical adsorption). Physical adsorption, also referred to as Van der Waals force, involved weak bonding between gaseous molecules and the solids. Chemisorption is a classification of adsorption characterized by a strong interaction between an adsorbate and a substrate surface, as opposed to physisorption which is characterized by a weak Van der Waals force. A distinction between the two can be difficult and it is conventionally accepted that it is around 0.5 eV of binding energy per atom or molecule. The types of strong interactions include chemical bonds of the ionic or covalent variety, depending on the species involved. Unlike physisorption mechanism, another distinguishing feature of chemisorption is that only a monomolecular layer of adsorbate appears on the adsorbing medium. This effect is due to the extremely short distances over which the valence forces holding the adsorbate to the adsorbent are effective. Based on the chemisorption mechanism, it is proposed that the free iron oxides are assumed to completely react with H_2_S and solid-phase product is iron sulfide. The assuming reactions for free iron oxides and H_2_S are described as follows:

(1) Fe2O3+2H2S+CO⟶2FeS+2H2O+CO2

(2) Fe3O4+3H2S+CO⟶3FeS+3H2O+CO2

(3) Fe2O3+2H2S+H2⟶2FeS+3H2O

(4) Fe3O4+3H2S+H2⟶3FeS+4H2O

**Figure 2:**
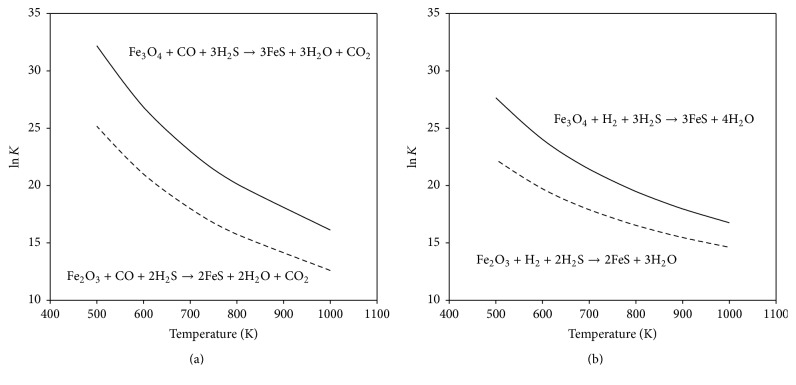
The thermodynamic calculation of the reactions between iron oxides and H_2_S at high temperature.

Prior to the experimental proceeding, thermodynamic calculations were simulated for the above reactions.  Figure 2 illustrates the equilibrium constants as a function of temperature for the reaction of iron oxides and H_2_S in the presence of CO and H_2_. Note that the equilibrium constants for the reaction of Fe_3_O_4_ and H_2_S are more favorable than that of Fe_2_O_3_ and H_2_S, which implies that the formation of FeS is easy to be achieved from Fe_3_O_4_ in the presence of CO and H_2_. On the other hand, the equilibrium constants are higher in the presence of CO, whereas lower equilibrium constants can be observed in the presence of H_2_. On the basis of this observation, it is expected that the CO may be a better gaseous reagent to promote the formation of FeS and a better estimated total free iron would be obtained in this study.

**Figure 3:**
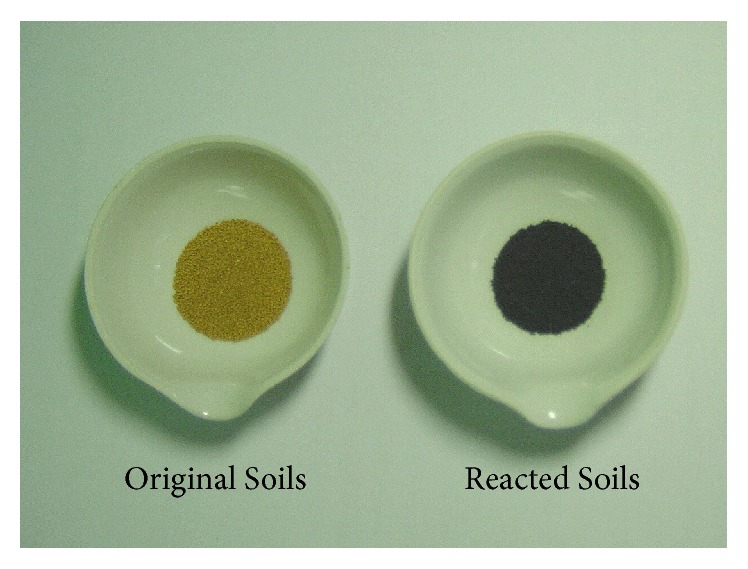
The photograph of Loupi soil before and after H_2_S chemisorption reaction.

It is very important to find out the optimal operation temperature for chemisorption because it is the key point to govern the overall performance for the reaction between free iron oxides and H_2_S in this system. Generally, low temperature cannot achieve the required performance, whereas elevated temperature causes the increase in experimental equipment cost. As shown in Table 1, Loupi series has the highest content of CBD-Fe among the representative samples. Therefore, Loupi series was chosen as a candidate to screen the optimal operation temperature. A series of temperatures ranging from 673 to 973 K with 50% of H_2_ and 1% H_2_S were conducted and experimental result was shown in Table 2. The breakthrough time increased with operation temperature and the optimal operation temperature was found at the temperature ranges of 723–823 K. In addition, note that the low temperature and higher temperature are not the best operation temperature in this study. Low temperature could not provide sufficient energy for reactions (1) and (2). On the other hand, under high temperature, the iron oxides are easy to be reduced to FeO in the strong reductive atmosphere. The formation of FeO leads to the weaker bonding with H_2_S to form FeS compared to Fe_2_O_3_. In order to confirm the presence of sulfur after reaction, the reacted soil sample was evaluated through an EA analyzer. Experimental results show that the sulfur contents determined from breakthrough time at various temperatures are close to these analyzed by EA, which indicated the excellent sulfur recovery for all operation temperatures. The change in color for Loupi soil sample before and after chemisorption is displayed in Figure 3. After chemisorption, the color of soil immediately turned into black. This implies that the reaction mechanisms for iron oxides and H_2_S are based on reactions (1) and (2).  Table 3 shows the effect of operating temperature on the sulfur capacities and estimated total free iron oxides for Loupi soil sample in the presence of CO and H_2_S. Similar results were observed for both CO and H_2_. The optimal operating temperature appeared to be at the temperature ranges of 723–823 K. Either low or high temperature does not achieve the optimal performance. In comparison to the estimated total free iron oxides that derived from the presence of H_2_ and CO, it can be found that the best operating temperature was controlled at 773 K and the highest estimated total free iron oxides are obtained. The estimated total free iron oxides are very close to the CBD-extracted total free iron oxides for the case of CO presence at 773 K. It is concluded that the operating temperature of 773 K and CO are the best operating condition in this study. Therefore, six soil samples were evacuated to determine their total free iron oxides at 773 K in the presence of CO and H_2_, respectively. As shown in Tables 4 and 5, the sulfur balance of various soil samples is found in the range of 88–102% under the experimental condition of 50% H_2_ and 1% H_2_S and better sulfur balances (94–103%) can be observed in the case of 50% CO and 1% H_2_S. The estimated total free iron oxides are lower than those obtained from CBD method for the experiment of 50% H_2_ and 1% H_2_S. On the other hand, the estimated total free iron oxides determined from the experiment of 50% CO and 1% H_2_S are close to those of CBD for all test soil samples. The estimated total free iron oxides of Taikon, Kuiren, Wukuiliao, and Houli are nearly corresponding to the CBD method, implying that the chemisorption is a potential technique to determine the total free iron oxides in soils and CO is the optimal candidate to achieve better experimental results in this study.

**Figure 4:**
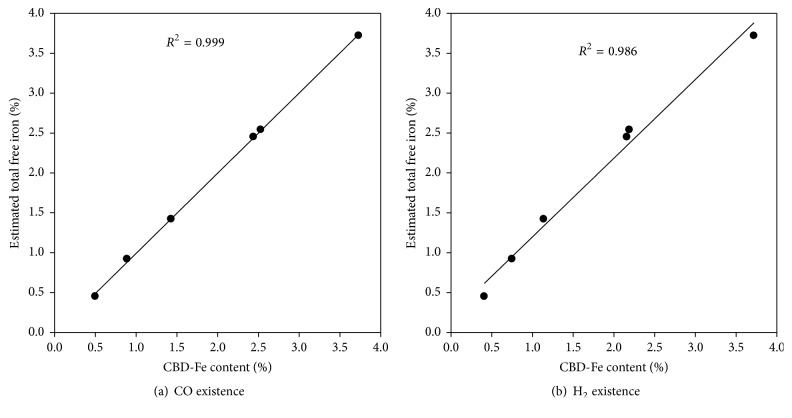
The regression relationship between CBD-Fe content and the estimated total free iron under the experimental condition of CO and H_2_, respectively.

Figure 4 shows the regression relationship between the CBD-Fe content and the estimated total free iron for six soil samples under the experimental condition of CO and H_2_. Both gases possess pretty good relationship with *R*^2^ values of 0.999 and 0.986, respectively, which indicated that they are a suitable reagent to determine the total free iron in soils.

## 4. Conclusions

An H_2_S chemisorption method based on thermodynamic knowledge was proposed for measurement of total free iron oxides in soils. Several representative soil samples were collected from Taiwan and assessed feasibility for measurement of total free iron oxides. Experimental results showed that the optimal operating temperature was found to be at 773 K for all soil samples. It was also found that carbon monoxide (CO) is the best gaseous reagent to promote the formation of FeS. The estimated total free iron oxides for soil samples determined from the chemisorption in the presence of CO were very close to those from the CBD technique.

## Figures and Tables

**Table 1:** Basic physical and chemical properties of the representative soil samples.

Soil series	Soil order	Soil group	pH	Sand	Silt	Clay	Texture	CEC (cmole kg^−1^)	CBD-Fe (Fe_2_O_3_%)	CBD-Mn (mg kg^−1^)
Taikon	Alfisol	Slate noncalcareous	8.4	6.5	46.2	47.3	Silt clay	20.5	2.54	427
		Older alluvial soils								
Kuiren	Alfisol	Noncalcareous	5.4	22.5	56.1	21.4	Silt loam	12.4	1.42	162
		Taiwan clays								
Wukuiliao	Inceptisol	Slate noncalcareous	7.1	13.9	60.3	25.8	Silt clay loam	14.2	2.45	923
		Older alluvial								
Sofong	Entisol	Schist calcareous	7.8	61.6	37.0	1.4	Sandy loam	5.7	0.45	142
		Younger alluvial soils								
Jensui	Entisol	Schist noncalcareous	5.7	65.6	31.8	2.6	Sandy loam	8.3	0.92	558
		Younger alluvial soils								
Houli	Ultisol	Diluvium red soils	5.1	32.2	35.6	32.2	Silt loam	7.8	3.72	245
Loupi	Ultisol	Diluvium red soils	4.0	14.3	34.2	51.5	Clay	8.40	4.28	117

**Table 2:** The sulfur capacities of the Loupi soil at various temperatures under the experimental condition of 50% H_2_ and 1% H_2_S with balanced N_2_.

Temperature	Breakthrough time (min)	Sulfur content (%)	Sulfur content by EA (%)	Sulfur balance (%)	Estimated total free iron (Fe_2_O_3_%)
673 K	20	0.22	0.23	95.65	2.05
723 K	26	1.13	1.12	100.89	2.66
773 K	32	1.39	1.37	101.46	3.28
823 K	30	1.31	1.30	100.76	3.07
873 K	21	0.91	0.92	98.91	2.15
923 K	19	0.83	0.84	98.81	1.95

CBD-Fe: 4.28 (Fe_2_O_3_%).

**Table 3:** The sulfur capacities of the Loupi soil at various temperatures under the experimental condition of 50% CO and 1% H_2_S with balanced N_2_.

Temperature	Breakthrough time (min)	Sulfur capacity (%)	Sulfur capacity by EA (%)	Sulfur balance (%)	Estimated total free iron (Fe_2_O_3_%)
673 K	30	1.31	1.32	99.24	3.07
723 K	41	1.78	1.77	100.56	4.19
773 K	42	1.83	1.81	101.10	4.30
823 K	40	1.74	1.72	101.16	4.09
873 K	35	1.52	1.50	101.33	3.58
923 K	33	1.44	1.44	100.0	3.38

CBD-Fe: 4.28 (Fe_2_O_3_%).

**Table 4:** The sulfur capacities and estimated total free iron of six representative soil samples at 773 K under the experimental condition of 50% H_2_.

Soil series	Breakthrough time (min)	Sulfur capacity (%)	Sulfur content by EA (%)	Sulfur balance (%)	Estimated total free iron (Fe_2_O_3_ %)	CBD-Fe (Fe_2_O_3_ %)
Taikon	20.1	0.88	0.89	98.88	2.19	2.54
Kuiren	10.5	0.46	0.50	92.0	1.14	1.42
Wukuiliao	19.8	0.86	0.84	102.38	2.16	2.45
Sofong	3.8	0.16	0.18	88.88	0.41	0.45
Jensui	6.9	0.30	0.31	96.77	0.75	0.92
Houli	30.7	1.34	1.37	97.81	3.34	3.72

**Table 5:** The sulfur capacities and estimated total free iron of six representative soil samples at 773 K under the experimental condition of 50% CO.

Soil series	Breakthrough time (min)	Sulfur capacity (%)	Sulfur content by EA (%)	Sulfur balance (%)	Estimated total free iron (Fe_2_O_3_%)	CBD-Fe (Fe_2_O_3_%)
Taikon	23.2	1.01	1.02	99.02	2.53	2.54
Kuiren	13.1	0.57	0.55	103.64	1.43	1.42
Wukuiliao	22.4	0.98	0.97	101.03	2.44	2.45
Sofong	4.6	0.20	0.18	94.44	0.50	0.45
Jensui	8.2	0.36	0.35	102.86	0.89	0.92
Houli	34.3	1.49	1.47	101.36	3.73	3.72
